# Barriers to accessing health care among undocumented migrants in Sweden - a principal component analysis

**DOI:** 10.1186/s12913-021-06837-y

**Published:** 2021-08-17

**Authors:** Hatem Mona, Lena M.C. Andersson, Anders Hjern, Henry Ascher

**Affiliations:** 1 School of Public Health and Community Medicine, The Institute of Medicine, Sahlgrenska Academy University of Gothenburg, Gothenburg, Sweden; 2 Department of Social Work, University of Gothenburg, Sprängkullsgatan 23, PO Box 720, SE- 405 30 Gothenburg, Sweden; 3 Clinical Epidemiology, Department of Medicine, Karolinska Institute, Stockholm, Sweden; 4 Centre of Health Equity Studies (CHESS), Karolinska Institute, and Stockholm University, Stockholm, Sweden; 5 Research Department, Angered Hospital, Gothenburg, Sweden

**Keywords:** Undocumented migrants, Access to healthcare, Sweden

## Abstract

**Background:**

Undocumented migrants face many hardships in their everyday life such as poor living conditions, discrimination, and lack of access to healthcare. Previous studies have demonstrated considerable health care needs for psychiatric disorders as well as physical diseases. The aim of this paper was to find out the main barriers that undocumented migrants experience in accessing the Swedish healthcare system and to explore their relation with socioeconomic factors.

**Methods:**

A cross-sectional study with adult undocumented migrants was performed in the three largest cities of Sweden in 2014–2016. Sampling was done via informal networks. A socioeconomic questionnaire was constructed including 22 barriers to health care. Trained field workers conducted the interviews. A principal component analysis was conducted of all barriers to reveal central components. Then, Pearson’s chi-squared test was used to explore the characteristics of undocumented migrants experiencing barriers to care.

**Results:**

Two main components/barriers were extracted: “*Fear of being taken by police/authorities*”, which was related to fear of disclosure by or in relation to seeking health care, and “*Structural and psychosocial factors*” which was related to practical obstacles or shame of being ill. Lower age (74.1 % vs 56.0 %), lower level of education (75.0 % vs. 45.1 %), and having no children (70.3 % vs. 48.1 %) were significantly related to a higher likelihood of experiencing a barrier.

**Conclusion:**

Fear of deportation and practical and psychosocial factors constitute hinderance of access to healthcare for undocumented migrants in Sweden. This highlights the importance of clear instructions, both to undocumented migrants and health professionals about the right to health care according to the international law on human rights as well as the law of confidentiality.

**Supplementary Information:**

The online version contains supplementary material available at 10.1186/s12913-021-06837-y.

## Background

Forced migration is a main feature of the world today with more than 70 million people, almost 1 % of the population on the globe, living as refugees as a result of forced migration. Large numbers of people flee their countries in search of refuge from violence and conflicts. A small number of them, 612,000, sought asylum in the European Union (EU) in 2019. The same year, over 60 % ended up with a rejection of their request for protection in the EU [1], and in Sweden, only 35 % of asylum decisions were granted [2]. Individuals with a failed asylum claim are expected to leave the country on a voluntary basis or risk to be deported. Some of them try to avoid this by remaining in the country clandestinely as undocumented migrants. The number of undocumented migrants in the EU is difficult to approximate. Most available figures are old, unsecure and are given with a wide range of variation. However, the statistical office of the European Union, Eurostat, estimates that in 2019, 627,900, undocumented migrants were residing in the EU [3].

Undocumented migrants are defined by the Health for Undocumented migrants and Asylum seekers (HUMA) as “a) persons who are planning to seek asylum but have not formally submitted an application to asylum to the national competent authorities; b) rejected asylum seekers (those asylum seekers whose application for asylum failed); c) persons whose application for residence permit/authorization to stay/family reunification is still pending (no decision has been taken by the competent authorities) even though in some countries they are considered to be in a regular situation; d) persons whose application for residence permit/authorization to stay/family reunification or renewal of this authorization has failed; e) overstayers of visas (e.g. tourist, student, medical reasons); overstayers of expired residence or work permits; g) persons who did not apply for any visa or residence permit and entered illegally” [4].

A recent review of the International Organization for Migration demonstrated that the universal human right to health care in many countries is limited for undocumented migrants [5]. In Sweden a law was passed in 2013 that gave undocumented migrants the right to health care subsidized for “care that cannot be deferred”. Prior to that, access was only allowed for emergency care but was unsubsidized, meaning that in practice care was inaccessible [6]. One study suggested that some EU states have the tendency to limit access to care services in order to discourage more migrants from seeking asylum [7]. Human rights, including the right to health, are formed to be enjoyed by all persons without discrimination. They are particularly directed to include disadvantaged groups including undocumented migrants [8, 9]. This is also in line with the Sustainable Development Goals (SDG) commitment to leave no one behind [10]. In addition to the human rights’ costs, excluding undocumented migrants from health care has been demonstrated to be economically costly [11]. With this in mind, whenever there are disparities in access related to socioeconomic factors, status of residence, or other personal traits, inequities arise [12]. Investigating problems of access to care for undocumented migrants is of great importance because EU countries have different regulations regarding access to care for this vulnerable group, and since research on this group remains scarce [13]. Also, little attention has been paid towards the integration of undocumented migrants in health programs, reflecting the political stances of many governments towards this group.

### Barriers to health care

Several reports on undocumented migrants in Europe discuss the importance of different barriers, such as a lack of knowledge about entitlement to care, fear of deportation, financial burdens to access secondary care, and cultural, administrative, and language barriers to communicate with health workers and negotiate treatment [5, 14–16]. In addition to those obstacles, undocumented migrant women face obstacles in accessing reproductive healthcare and being subjected to physical and sexual violence [17–19]. Compared to documented ethnic minority women, undocumented pregnant women in the Netherlands were also more likely of giving birth at home, and received their first clinical consultation five weeks later than the control group [20]. When faced with mental illnesses, undocumented migrants in the Netherlands prioritized first contacting friends and relatives for consultation, while placing the general practitioner last [21]. In Denmark, undocumented migrants´ experiences of access to healthcare showed barriers relating to lack of knowledge about informal networks of health professionals and the healthcare system overall, lack of social networks with native Danes, fear of deportation by authorities, and language barriers. This resulted in undocumented migrants seeking alternative strategies for treatment, like self-medication [22]. At the same time, it should be made clear that different regulations for the right to care apply differently for undocumented migrants in Europe, hence, ambiguities surrounding what is defined as emergency care “contributed to uncertainty among health professionals” [23–26]. In spite of that, undocumented children have, according to the law, equal access to health care, dental care and preventive care as Swedish resident children. However, questioning the parents, especially when asking for identification, was a main obstacle to child immunization for undocumented migrants in Sweden [27].

### Large need for health care and risk factors for illness

Undocumented migrants live in very difficult social circumstances in Sweden [28] including an inability to work legally, rent an apartment [29], pay expenses and having food shortages [30]. Moreover, war related factors also contribute to their vulnerability such as past traumatic experiences of war, violence, abuse and post-traumatic stress syndrome (PTSD), uncertainty about the future, chronic stress due to the risk of disclosure, and deportation, often to a location connected to previous traumatic experiences. Work opportunities for this group is usually short term and underpaid. Many are separated from their families and have difficulty securing finances for themselves [31]. A survey of pregnant undocumented migrants in Geneva, a particularly vulnerable subgroup, found that four out of five pregnancies resulting in live births were unintended [32]. Another study in Switzerland on undocumented migrants in primary care showed that 72 % of the participants had at least one chronic physical condition [33].

The burden can become greater when undocumented migrants need to provide for their dependents. A Norwegian study showed high levels of psychological distress associated with experiencing abuse, and poor living conditions, like hunger and homelessness. An interesting finding was that there was no statistical significance between presence of family members and reduction of stress. Indeed, having a family meant that any positive effects of the family on the individual regarding their mental health, was outweighed by the much-increased responsibilities to sustain the family [34]. When comparing documented and undocumented migrants, differences in healthcare utilization arise. A systematic review by Winter et al. [13] found lower utilization rates of primary healthcare and lower number of consultations with general practitioners when comparing undocumented migrants with regular migrants, and further that care was insufficient even when provided. Similarly, a Dutch study looking at medical records compared undocumented and documented migrants and found that undocumented migrants consulted their general practitioner significantly less than the reference group, 3.24 times a year versus 5.04 [35].

Many barriers to care have been presented in the literature. However, for policy makers to make use of these evidence-based findings, there is a need to identify main themes in those barriers that emerge in order to design adequate interventions. In this study we used data from the Swedish SHERUM project, one of the largest studies in Scandinavia of undocumented non-patient migrants, investigating psychological, social and health related factors. In this paper we conducted a Principal Component Analysis (PCA) in order to identify main themes and components of barriers to health care that were experienced by the undocumented migrants and a chi-squared test to find the relation between socioeconomic factors and experiencing barriers.

## Method

### Study design and population

The study design was cross sectional and the data was collected from survey interviews with undocumented migrants in informal health clinics (a clinic administered by voluntary organisations that are not part of the formal public health system) in the largest three cities of Sweden: Stockholm, Gothenburg, and Malmö during the period 2014–2016 [30]. Inclusion criteria were age > 18 years and status as undocumented, either due to rejected asylum application, overstaying the visa period, or moving to Sweden without a visa in the first place. EU citizens that overstayed their visa duration were excluded.

### Sampling

Convenience population-based sampling was used due to the difficulty of recruiting undocumented migrants who commonly fear disclosure and deportation. The Rosengrenska clinic in Gothenburg, Doctors of the World in Stockholm, and the Red Cross in Malmö and Stockholm as well as religious institutions and civil society were crucial in getting in contact with the undocumented migrants. Such networks provide undocumented migrants with clothes, food, and health services and acted as a meeting place for support in the community. Most participants in the study went to the clinic due to food distribution and social activities, and not primarily for physical or mental health problems.

### Questionnaire and instruments

A questionnaire was developed to investigate undocumented migrants´ living conditions and health. It consisted of 112 multiple-choice questions. Topics covered were background, access to food, living conditions, education, income, physical and mental health, barriers to care, social networks, access to care. Several of the questions concerning access to health care and barrier to care was developed in an earlier study by Andersson et al. [36]. It was developed based on questions that concerned health care use and barriers to care that was used in several previous studies [37–44]. Right to health questions were constructed based on the CESCR General Comment No.14: The Right to the Highest Attainable Standard of Health (Art.12) [8]. Three instruments were used for screening depression (Beck’s Depression Inventory BDI-II), anxiety (Beck’s Anxiety Inventory BAI), and Post traumatic stress syndrome (PTSD, PCL-5). Self-rated questions on mental and physical health were also included. The principal investigator (H.A) obtained license to use the Beck’s Depression Inventory BDI-II, Beck’s Anxiety Inventory BAI and PCL-5 in this project: the Swedish Health Research on Undocumented Migrants Project (SHERUM).

### Semi structured interviews

No incentives were offered to the participants when they were asked to participate in the study. Translators were used both during recruitment and during most interviews. Interviews took place in churches and voluntary organizations according to the choice of the respondents. The interviews took approximately 1–1,5 h and were done with the use of an iPad/tablet in which the questionnaire was placed. The questionnaire was translated into nine languages. However, since most respondents did not speak either Swedish or English, translators were used in the majority of interviews. Some interviews were done at two occasions at the request of the respondent. Psychological counselling was offered if the respondent needed it after the interview. Five respondents felt such a need. In total 104 participants were recruited; 54 men and 50 women [30].

### Ethical considerations

The Swedish Central Ethical Review Board in Gothenburg, Sweden approved the study (ref no. Ö 25-2013). Participants were told that no direct intervention from the study would assist them in their asylum cases, and their right to refrain from answering any question they didn’t want to answer or withdraw from the study at any time. The interviewers had psychosocial skills to handle vulnerable individuals during conversations and possessed adequate information on referring participants to health staff or counsellors when needed. Respondents received a code number to secure anonymity. Verbal consent was used instead of written consent, so that participants would be assured their names and any evidence of their participation was kept confidential.

### Statistical analysis

Since the focus in this study was on barriers to health care, we wanted to be sure that the interviewees included in the study population had had needs to access health care while being undocumented migrants. Thus, the 12 interviewees who responded “No” to a question about having had health problems in Sweden that needed health care were excluded from the study, leaving 92 interviewees in the final study population. All statistical analyses were conducted in SPSS version 25.0 (IBM SPSS Statistics for Windows, Version 25.0. Armonk, NY: IBM Corp.)

A summarised dichotomised variable of “ Having experienced barriers to health” was created based on 22 single items in the questionnaire. Characteristics of interviewees that had experienced such barriers were explored in a chi-square analysis with the calculation of Pearson chi-square coefficients. No multivariate analyses were performed because of the considerable internal attrition on many items. Principle component analysis was performed to reduce the 22 items of barriers into components by measuring correlations. The barriers that were correlated were grouped under the same components to provide meaningful themes for barriers. In this study, the components obtained represented obstacles of accessing healthcare experienced by the undocumented migrants in the study. Twenty-two barriers were included in the questionnaire. The Kaiser-Meyer-Olkin Measure of Sampling Adequacy (KMO) statistic indicated that the data was suitable to conduct PCA (0.708). Any number greater than 0.5 for this test shows that PCA can be conducted. The Bartlett’s test of sphericity significance tests for statistical significance was 0.000. A value less than 0.05 in this test rejects the null hypothesis, which is that no barriers were correlated with any other barriers (an identity matrix).

The first step in the PCA was to choose the number of components to extract, which depends on the author’s judgement. Especially for the case of this sample having a small sample size, a “theory with fewer factors is usually preferable” [45]. Those with a value greater than 0.3 for one component and less than 0.3 for the other were grouped under the former component to establish that component. However, barriers that had values greater than 0.3 for both components after rotation were also excluded because it would not be possible to assign them to a component based on collinearity. This means those barriers had enough collinearity to be related to both components [45].

## Results

The sample for this study consisted of the 92 individuals who answered yes on the question: ”Have you ever felt so ill that you needed care?”. Their characteristics are presented in Table 1. The time that the interviewees had lived as undocumented in Sweden varied considerably with 28 % having been in this situation for less than a year and 35 % for more than two years. As many as 35 % had a university education in the country of origin while 38 % had no more than a primary education. Morbidity levels were high with 62 % reporting a chronic physical illness, 71 % suffering from a moderate or severe depression, 68.8 % from moderate to severe anxiety, and 52.9 % from PTSD according to the instruments used in the interview [30].

**Table 1:** Sociodemographic characteristics and morbidity indicators of the study population (*N* = 92)

		n	Valid %
***Sociodemographic indicators***			
Sex	Male	47	51.1
	Female	45	48.9
Age (years)	18–23	19	20.7
	24–28	12	13.0
	29–34	18	19.6
	35–80	40	43.5
	Missing	3	
Civil status	Not in a relationship	50	56.8
	In a relationship	38	43.2
	Missing	4	
Education	Primary	23	27.7
	Lower secondary	9	10.8
	Upper secondary	22	26.5
	+ University	29	34.9
	Missing	9	
Year as undocumented migrant	0–12 months	25	27.5
	13–24 months	34	37.4
	2 years or more	32	35.2
	Missing	1	
Have children	No	37	41.6
	Yes	52	58.4
	Missing	3	
***Morbidity***			
At least one chronic physical illness	No	35	38.5
	Yes	56	61.5
	Missing	1	
Depression	No or mild depression	19	28.8
	Moderate or severe depression	47	71.2
	Missing	26	
Anxiety	No/mild to moderate	24	31.2
	Moderate to severe	53	68.8
	Missing	15	
PTSD	No	24	47.1
	Yes	27	52.9
	Missing	41	

In the study population, 51 (55.4 %) reported at least one barrier accessing healthcare. The younger interviewees, those with no children, and interviewees with no more than a lower secondary education more often reported having experienced such barriers (Table 2).

**Table 2:** Having experienced barriers in accessing health care in Sweden as an undocumented migrant by covariates. Row percentages

		n	N(%)	*p*-value
***Sociodemographic indicators***				
Sex	Male	47	29 (61.8)	*p* = 0.216
	Female	45	22 (48.9)	
Age (years)	18–28	31	23 (74.1)	***p* = 0.019**
	29–80	50	28 (56.0)	
Civil status	Not in a relationship	50	32 (64.0)	p = 0.119
	In a relationship	38	18 (47.3)	
Education	Primary + Lower secondary	32	24 (75.0)	***p* = 0.007**
	Upper secondary + University	51	23 (45.1)	
Year as undocumented migrant	0–24 months	59	37 (62.7)	*p* = 0.082
	2 years or more	32	14 (43.8)	
Have children	No	37	26 (70.3)	***p* = 0.037**
	Yes	52	25 (48.1)	
***Morbidity***				
At least one chronic physical illness	No	35	22 (62.9)	*p* = 0.301
	Yes	56	29 (51.8)	
Depression	No or mild depression	19	10 (52.7)	*p* = 0.843
	Moderate or severe depression	47	26 (55.3)	
Anxiety	No/mild to moderate	24	15 (62.5)	*p* = 0.429
	Moderate to severe	53	28 (52.8)	
PTSD	No	24	13 (54.2)	*p* = 0.714
	Yes	27	16 (59.3)	
* All*		*92*	*51 (55.3)*	

### The principal component analysis

Supplementary Table 1 shows the total variance in the sample explained by the first 7 components along with the eigenvalues assigned to each component (See supplementary Table S1). These eigenvalues are also presented in the scree plot (See Supplementary Figure S1). In the scree plot, the points before a shift in the slope are taken as components. The second point on the graph is the last point before the slope becomes somewhat levelled, thus, this data will comprise of two components. After that, the rotation component matrix (Table 3) was checked for correlations between the two components and the barriers.

**Table 3:** Rotated component matrix showing the loading values of each barrier for each component

Barriers	Component 1	Component 2
I was afraid the police would take me when I was at the hospital/clinic	0.867	0.031
I was afraid of all the questions the personnel would ask me	0.662	-0.007
I head about another UM that was rejected	0.407	0.436
I have a bad experience of how staff responded to me on previous occasions	0.097	0.178
I was afraid someone would see me when I seek the clinic and call the police/ migration authorities	0.88	0.189
I became discouraged of another UM experience and what they saw	0.677	0.323
I was afraid to go to the hospital due to the risk of being reported to the Swedish authorities	0.81	0.161
It was too far for me to get there	0.227	0.181
I was afraid the police would take me so I travelled to the informal caregiver	0.575	0.339
I could not afford to pay the healthcare fee	0.393	0.74
I had no one interpreting for me	0.453	0.484
There was no public transport so I could not get there	-0.014	0.518
I could not pay the patient fee	0.461	0.479
I thought the waiting times were too long	0.074	0.737
I did not know where I could go to get healthcare	0.434	0.437
I did not feel I needed medical care for this problem	-0.003	0.679
I did not think I could seek medical care	0.522	0.208
I did not think care would help me	0.29	0.364
I thought I could handle the problem myself	0.164	0.265
I could not tell my problems	0.337	0.484
I was afraid someone I knew would realise that I was sick	0.111	0.498
I did not think the personnel would keep quite about my ill health in front of others	0.549	0.18

From the 22 barriers, 10 satisfied the requirements from the loading values and were included under the two components. Table 4 shows the loaded barriers under the components.

**Table 4:** Barriers loaded onto the two components that are named

Component 1: *Afraid of being taken by the police/authorities*.	Component 2: *Structural and psychosocial factors*
Afraid the police would take me at the clinic/hospital	There was no public transport, so I could not go there
Afraid of questions the staff would ask me in the clinic/hospital	I thought the waiting time was too long
Afraid of being seen while going to the clinic and having someone call police/authorities	I did not feel I needed medical care for my problem
Afraid of going to the clinic due to the risk of being reported to police/authorities while there	Afraid someone I knew would realize that I was sick
I did not think I could seek medical care	
I did not think the staff would keep confidentiality about my ill health	

Two components were identified in the analysis and are presented in Table 4; Component #1 was labelled *Fear of being taken by the police/authorities* and Component #2 *Structural and psychosocial factors.* In Component #1, items that indicated *Being Afraid’* appeared in four out of six items. The last item under component 1 was named *“Fear”* as having no belief in the staff keeping confidentiality can also lead the staff to report patients to authorities. Consequently, fear of being reported to or taken by the police or authorities is the main theme reflected by these barriers. The only exception to this theme was the barrier “I did not think I could seek medical care”, which could have been chosen for several reasons. For example, it could be the case that some undocumented migrants did not know they were entitled to receive medical care.

Component #2, *Structural and psychosocial factors*, has four items. Two items relate to practical factors; lack of public transport to travel to a clinic and long waiting times before meeting the doctor. The other two barriers relate to the way a patient perceives illness and the way their family and relatives interact with their illness. The feeling that the undocumented migrant does not need medical care can stem from not feeling sick in the first place, or from not feeling that the case needs medical attention. Being afraid of acquaintances finding out about a disease could be a consequence of cultural perceptions on certain illness or conditions like depression and anxiety.

## Discussion

This study investigated barriers for accessing health care in a convenience sample of undocumented migrants in the three largest cities in Sweden during 2014–2016. A little more than half of the interviewees reported having experienced such barriers, with those with lower age (age group 18–28 versus age group 29–80), (74.1 % vs. 56.0 %), lower educational attainment (primary + lower secondary school versus upper secondary school + university) (75.0 % vs. 45.1 %), and those having no children (having no children versus having one or more), (70.3 % vs. 48.1 %) being more likely to report having experienced barriers to care. The Principal Component Analysis revealed two different types of barriers: *‘Fear of being taken by the police/authorities`* and *‘Structural and psychosocial factors’*.

### Fear is an important factor for not seeking health care

The first component ‘*Fear of being taken by the police/authorities*’ explained 23.7 % of the variance after varimax rotation making it the most prominent component. Fear of disclosure by the police or migration authorities leading to deportation was the main concern for not contacting health clinics. This is in accordance to the findings in other studies from Europe [14–17, 22]. With regard to experienced fear, trauma obtained during pre-migratory and migratory periods often remains after undocumented migrants arrive in their host countries [46]. Witnessing war and death of family members and relatives as well as the journey of escaping home are all relevant reasons for not having a feeling of general and self-security. This lack of security generates fear of being returned home to the place of conflict. Furthermore, the fear that health care staff will break the laws of patient confidentiality and contact the police and authorities, as found in this study, is widely spread among undocumented migrants. Probably this is an effect of the high level of fear of being deported back to a place associated with previous traumatic events. However, it seems to be an unusual occurrence in Sweden that health care staff report to the police. Much more common is that undocumented migrants are wrongly denied health care they are entitled [25]. This in turn is likely to be an effect of complicated formulation of the law. According to the preparatory work of the law, more or less all types of health care are included in the concept *health that could not be deferred*. This is however not a widespread knowledge among health professionals [25, 26].

### Structural and psychosocial factors affect undocumented migrants´ help seeking behaviour

Component 2 *“Structural and psychosocial factors”* explained 17.1 % of the variance in the sample. Since four barriers were included in this component, it was given a name encompassing two different themes. The structural part pertains to long waiting times to access a clinic and no public transport. In Sweden, public clinics follow a queue system, so a patient willing to visit the clinic must, unless the condition is acute, often wait for hours, or some days before meeting the doctor. Such system may discourage undocumented migrants to visit the public clinic when their conditions do not seem serious. This is also found in other migrant groups [47]. In some studies, high cost is a common barrier to care [15, 16], whereas in Sweden, the cost of healthcare for undocumented migrants is subsidized according to the law [48]. Despite that, the cost is too high for many undocumented migrants who usually live under strained economic conditions. Moreover, lack of, or costs for public transport was a barrier in this study.

The psychosocial part in this construct relates to ‘Being afraid of acquaintances finding out about the disease’ and ‘Feeling that care is not needed’. Both barriers can be affected by the health literacy of undocumented migrants. Even though 61.4 % of the sample had a university or secondary high school level education, health literacy might not be reflected by the level of general education. Moreover, traditional views can play a role in the understanding of diseases, especially mental health ones. High levels of depression, anxiety, and PTSD are noted in the results indicating 71.2 %, 68.8 %, and 52.9 % respectively [30]. Furthermore, the barrier ‘*Feeling that care is not needed’* can be explained in two different ways. One way is that many undocumented migrants might have faced emotional and psychological events prior to arriving in Sweden due to war, persecution, torture, etc. Going through such events and consequently arriving to a stable country might create a sense of wellness. Nonetheless, after becoming undocumented, mental symptoms may be perceived by undocumented migrants as an effect of circumstances rather than a mental disorder, hence creating the feeling that care is not needed. Another could be traditional views. It is important to note that countries especially outside the EU follow different health systems and priorities in their healthcare system, and mental health is not always on top of the agenda [49]. In addition, mental health is in many parts of the world regarded as a stigma or a form of disability instead of a curable disease. This may affect the willingness to seek health care for mental ill-health and thus work as a barrier [50].

### Socioeconomic factors associated with having barriers to care

Unlike other studies that have found that age was not a predictor of barriers [14, 35], it was found in this sample that younger undocumented migrants (age 18–28) were more likely to experience any barrier (74.1 % compared to 56.0 %) than older undocumented migrants (aged 29–80). Similarly, undocumented migrants with primary or lower secondary education were more likely to report at least one barrier (75.0 % compared to 45.1 %) than undocumented migrants with upper secondary or university education. No previous studies regarding undocumented migrants showed a relation between education and barriers, perhaps due to the nature of the studies, since most of them were qualitative or had small sample sizes. One study however, illustrated that after multivariate analysis, lower levels of education were associated with lower utilization of healthcare services [51]. One may speculate that having higher education facilitates the search for accessible health care in the new environment. Moreover, results showed that undocumented migrants with no children were more likely to have barriers (70.3 % compared to 48.1 %) than those with children. In Sweden, undocumented children have the same rights to health care as resident children. It seems possible that some interviewees included their experiences of accessing care for their children in their response to this question. Furthermore, two studies by Wolf et al. [31, 32] found that undocumented migrants with dependents had their economic situation worsened since they were responsible to support their families financially, with food, education, and other needs, while having difficulties to sustain themselves with housing and meagre earning jobs. Undocumented migrants face different burdens compared to other migrant groups, as they are not entitled to public financial support.

### Limitations

This study has some limitations. Small sample sizes create the problem of asserting representability of the general population. However, the size of the sample is small due to the difficulty of recruiting members of this specific vulnerable group where research is scarce. Moreover, the strategy employed to recruit participants from the informal clinics was chosen out of the experience that many undocumented migrants, due to fear of disclosure, are afraid to take part in research studies. Also, it cannot be asserted that a representative sample of undocumented migrants living in the three mentioned cities actually go to these clinics, so the sample recruited might not represent the larger group of undocumented migrants with all their diversity. Another limitation was that those who answered the question of having any barriers were all participants that answered a prior question indicating they had not sought healthcare in Sweden before when they felt ill. This excluded persons who have gone at least once to a health clinic and due to a negative experience, stopped going back to the clinic afterwards. This means the barriers extracted from the PCA do not reflect barriers due to personal experiences at the clinic, but only those of participants who have not gone even once to the clinic. Lastly, cross-cultural validity for the measures cannot be guaranteed in this culturally diverse sample of participants, recall bias may be present, especially among those who suffered from PTSD and interview bias may also influence the results since we had several interviewers and translators.

## Conclusions

This study showed that undocumented migrants in Sweden face two main barriers in accessing healthcare services. The first is fear of being disclosed and deported by authorities and the second is structural and psychosocial factors which hinder undocumented migrants in reaching health care given the difficult living and work environments. These barriers result in many undocumented migrants resorting to informal health clinics instead of public health ones. However, these clinics, do not provide health care themselves, but sort out health problems, give help to seek care at the right place, inform undocumented migrants about their rights and try to mediate when undocumented migrants are wrongly rejected care. Therefore, much work is needed by the regions responsible for Swedish health care to improve access to care for undocumented migrants.

### Recommendations

In order to improve access to healthcare for undocumented migrants and build on the findings of this study, the Swedish health authorities should implement solutions to include undocumented migrants in the Swedish healthcare system, mainly by training and education of health care workers about the legal and human rights of undocumented migrants, but also of undocumented migrants themselves as rightsholders of their right to health care. From a human right’s perspective, and particularly in a right to health perspective, a formal or informal agreement should be made between migration and health authorities, emphasising that health care facilities in Sweden be regarded as safe zones for undocumented migrants so that they can seek and receive the care they are entitled to without fear. In addition, voluntary organisations relating to undocumented migrants should be supported and better funded to strengthen the social networks around undocumented migrants and enhance their health literacy to improve their resilience to illness. Social support and social capital also increase the likelihood of actual help seeking when needed.

## Supplementary Information

**Additional file 1: Supplementary Figure S1. **Scree plot showing the components and their eigenvalues.

**Additional file 2: Supplementary Table S1. **Total variance explained by initial eigenvalues and after varimax rotation

## Data Availability

The data that support the findings of this study is available and under the responsibility of the Department of Epidemiology and Social Medicine, at the Institute of Medicine, Sahlgrenska Academy, University of Gothenburg but restrictions apply to the availability of these data, which were used under license for the current study, and so are not publicly available. Questions or requests concerning this data is directed to the principal investigator Henry Ascher.

## Abbreviations

SHERUM: The Swedish Health Research on Undocumented Migrants Project
EU: European Union
SDG: Sustainable Development Goals
PCA: Principal Component Analysis
BDI-II: Beck’s Depression Inventory
BAI: Beck’s Anxiety Inventory
PTSD: Post-traumatic stress syndrome
CESCR: Committee on Economic Social and Cultural Rights
