# Supplementary material for: Barriers to accessing health care among undocumented migrants in Sweden - a principal component analysis
Source: BMC Health Serv Res. 2021 Aug 17;21:830. doi: 10.1186/s12913-021-06837-y (PMC8369752; doi:10.1186/s12913-021-06837-y)
Supplement: Supplementary file 1 — Additional file 1: Supplementary Figure S1. Scree plot showing the components and their eigenvalues. [file 12913_2021_6837_MOESM1_ESM.docx]

**Supplementary figure S1:** Scree plot showing the components and their eigenvalues
