# Supplementary material for: Barriers to accessing health care among undocumented migrants in Sweden - a principal component analysis
Source: BMC Health Serv Res. 2021 Aug 17;21:830. doi: 10.1186/s12913-021-06837-y (PMC8369752; doi:10.1186/s12913-021-06837-y)
Supplement: Supplementary file 2 — Additional file 2: Supplementary Table S1. Total variance explained by initial eigenvalues and after varimax rotation [file 12913_2021_6837_MOESM2_ESM.docx]

**Supplementary table S1:** Total variance explained by initial eigenvalues and after varimax rotation

| Component | Initial Eigenvalues | | Rotated sums of squared loadings | |
| --- | --- | --- | --- | --- |
|  | Total | % of variance explained | Total | % of variance explained |
| 1 | 7.601 | 34.548 | 5.214 | 23.698 |
| 2 | 2.371 | 10.779 | 3.772 | 17.147 |
| 3 | 1.796 | 8.162 |  |  |
| 4 | 1.347 | 6.123 |  |  |
| 5 | 1.236 | 5.618 |  |  |
| 6 | 1.225 | 5.568 |  |  |
| 7 | 0.895 | 4.070 |  |  |
